# N‑Terminal Pro-B-Type Natriuretic Peptide (NT-proBNP) as a Biomarker in Heart Failure With Preserved Ejection Fraction (HFpEF) Versus Heart Failure With Reduced Ejection Fraction (HFrEF): The Way Forward in the Age of Proteomics

**DOI:** 10.7759/cureus.94162

**Published:** 2025-10-08

**Authors:** Shweta Kanyal, Abhirami Das, Anas M Din Bashir, Ahmed H Syed, Savvy Aujla, Jainam Chaudhary, Dharmik Patel, Ashish Goel

**Affiliations:** 1 Department of Medicine, Lokmanya Tilak Medical College, Mumbai, IND; 2 Research Fellow, Pear Research, Mumbai, IND; 3 Department of Medicine, Rajiv Gandhi Medical College, Thane, IND; 4 Department of Medicine, Shalamar Medical and Dental College, Lahore, PAK; 5 Department of Internal Medicine, Shalamar Medical and Dental College, Lahore, PAK; 6 Department of Medicine, Government Medical College, Amritsar, IND; 7 Department of Medicine, GMERS Medical College, Himmatnagar, IND; 8 Department of Physiology, Government Doon Medical College, Dehradun, IND

**Keywords:** biomarkers, ejection fraction, heart failure, hfpef, hfref, nt-probnp, prognosis

## Abstract

Heart failure (HF) encompasses a spectrum of clinical syndromes with substantial global morbidity and mortality, primarily linked to coronary artery disease. Affecting over 37 million individuals worldwide, its prevalence increases with age. HF is classified into three major categories: heart failure with reduced ejection fraction (HFrEF), heart failure with preserved ejection fraction (HFpEF), and the intermediate phenotype, heart failure with mid-range ejection fraction (HFmrEF). Despite similar symptomatology, differentiating HFpEF from HFrEF remains challenging, particularly through biomarker evaluation. The diagnostic and prognostic roles of B-type natriuretic peptide (BNP) and N-terminal proBNP (NT-proBNP) are well established; however, an unmet need persists in accurately distinguishing HFpEF from HFrEF, as NT-proBNP levels are often influenced by age, renal function, and obesity, which confound its interpretation in HFpEF.

This review uniquely integrates current evidence delineating the biomarker’s pathophysiological underpinnings, clinical thresholds, and predictive capacities across HF phenotypes, while contrasting its limitations and interpretive nuances in real-world practice. For instance, while elevated NT-proBNP levels in HFrEF strongly correlate with ventricular dysfunction severity and guide therapy initiation, their diagnostic specificity in HFpEF is reduced, often necessitating adjunctive imaging or additional biomarkers for confirmation. Advances in proteomics are reshaping biomarker discovery, enabling the identification of novel cardiac stress markers such as soluble ST2 and growth differentiation factor-15 (GDF-15), which complement NT-proBNP by reflecting distinct molecular pathways of myocardial remodeling and inflammation.

Although NT-proBNP remains the gold standard for prognostic assessment and risk stratification in HF, its diagnostic performance, particularly in HFpEF, must be interpreted within the broader biomarker landscape. Future directions should emphasize proteomic integration and multimarker strategies to achieve precision-based HF phenotyping and improved therapeutic outcomes.

## Introduction and background

Heart failure (HF) presents as a combination of symptoms, such as breathlessness, lower limb swelling, and fatigue, along with observable signs like elevated jugular venous pressure, edema in dependent areas, and crackles upon auscultation [1]. The prevalence of HF in developing countries is on the rise, with coronary artery disease being the most common culprit [2]. It affects more than 37.7 million people worldwide and is often referred to as a pandemic, conferring a huge burden on the healthcare system [3]. The prevalence of HF in the Western population typically ranges from 1% to 2% [4], although it tends to be significantly higher among the elderly, with a median prevalence of 11.8% [5]. Among individuals below 40 years old, the prevalence is approximately 1%, while for those above 80 years old, it increases to around 10%. Between these age groups, both genders face a similar lifetime risk of approximately 20% [6].

Taking into account the left ventricular ejection fraction (LVEF), HF can be of three types: heart HF with reduced ejection fraction (HFrEF; EF <40%), HF with preserved ejection fraction (HFpEF; EF ≥50%), and the intermediate phenotype, HF with mid-range EF (HFmrEF; EF 40-49%) [7]. HFmrEF is increasingly recognized as a transitional or mixed phenotype, often sharing features of both systolic and diastolic dysfunction, with therapeutic and prognostic characteristics that justify its inclusion as a distinct clinical entity. Pathophysiologically, diastolic dysfunction in HFpEF arises from comorbidity-driven systemic inflammation and myocardial fibrosis, whereas HFrEF primarily results from direct cardiomyocyte loss due to ischemic or valvular injury, leading to systolic dysfunction. These mechanisms trigger neurohormonal activation, including the renin-angiotensin-aldosterone system (RAAS) and sympathetic pathways, causing cardiac hypertrophy and dilation that manifest as typical HF symptoms [8].

HF has an alarmingly high all-cause mortality at one year of around 8.1% [9], highlighting the repercussions of a delay in diagnosis. This delay can be attributed to a vague initial presentation, leading to misdiagnosis and deferred treatment, which worsens symptoms, increases hospitalizations, and contributes to comorbidities such as depression [10]. Prolonged congestion can exacerbate myocardial damage and heighten the risk for multiorgan dysfunction [11]. An episode of acute HF with pulmonary edema can cause severe respiratory distress as well as marked anxiety in the patient due to a feeling that is akin to ‘drowning’ [12]. Framingham, Boston, and Duke criteria for HF, which use clinical findings such as patient history, physical examination, and chest X-ray, have limited sensitivity (50-73%) and specificity (54-78%). The advent of noninvasive imaging such as echocardiography has improved diagnostic accuracy [13]. However, high costs and the presence of asymptomatic LV dysfunction underscore the need for novel diagnostic approaches [14].

Thanks to advancements in molecular cardiology, proteomics has become central to biomarker discovery, enabling the identification of proteins involved in myocardial stress, remodeling, and neurohormonal activation. These approaches have contributed to the evolution of established biomarkers such as B-type natriuretic peptide (BNP) and N-terminal proBNP (NT-proBNP), and facilitated the development of novel candidates like mid-regional pro-adrenomedullin (MR-proADM), growth differentiation factor-15 (GDF-15), galectin-3, and soluble ST2 (sST2) [15].

BNP and NT-proBNP, though closely related, differ in biological behavior: BNP is the active hormone with a shorter half-life and rapid clearance, while NT-proBNP is an inactive fragment with a longer half-life and greater stability, making it more clinically preferred for diagnostic use. Both are released from ventricular myocytes in response to increased wall stress and volume overload, reflecting myocardial strain. NT-proBNP levels rise proportionally with the severity of HF and decline with effective treatment, thus serving both diagnostic and prognostic purposes. Clinically, measurement is performed using standardized immunoassays, and interpretation is guided by age- and context-specific cut-off values to distinguish acute from chronic HF. Both serve as reference biomarkers in HF evaluation.

The EMPEROR-Reduced randomized trial established a strong association between NT-proBNP levels and rates of HF hospitalization and cardiovascular death [16], while large observational cohorts such as the Breathing Not Properly Study demonstrated the diagnostic value of BNP and NT-proBNP in relation to NYHA functional class [17]. Although NT-proBNP is widely regarded as a gold standard biomarker for acute HF diagnosis and prognostic stratification, controversy persists regarding its validity as a surrogate endpoint in HF trials.

The primary aim of this review is to emphasize the diagnostic and prognostic roles of BNP and NT-proBNP in HF, focusing on their clinical interpretation across HF phenotypes. Secondary objectives include examining the influence of medications and comorbidities on NT-proBNP and highlighting limitations and future perspectives in biomarker-guided HF management.

## Review

Methodology

This manuscript is structured as a narrative review, providing a comprehensive, expert-led synthesis of the current literature rather than an exhaustive systematic analysis. Consequently, we have not adhered to the formal requirements of the Preferred Reporting Items for Systematic Reviews and Meta-Analyses (PRISMA) statement, which are designed for systematic reviews. Our literature search was conducted up to August 2025 using PubMed, Google Scholar, and Web of Science. The primary search terms included 'NT-proBNP,' 'BNP,' 'heart failure,' 'HFpEF,' 'HFrEF,' 'biomarker,' 'prognosis,' 'therapy,' and 'limitations.' The final selection of studies, meta-analyses, and expert guidelines was based on their direct relevance to the role of NT-proBNP across different heart failure phenotypes. 

Selection Criteria

Articles were selected based on their clinical relevance, currency, and direct focus on NT-proBNP's role in the diagnosis, prognosis, and therapeutic guidance of HF. The included literature primarily consists of landmark randomized controlled trials, recent meta-analyses, expert consensus documents, and relevant guidelines from major cardiology societies (e.g., American College of Cardiology (ACC) and the American Heart Association (AHA), European Society of Cardiology (ESC). Reviews and observational studies with high clinical impact were also included for contextual and mechanistic information.

Reporting Limitations

Consistent with the nature of a narrative review, we did not perform a formal, quantitative risk of bias assessment for individual studies (e.g., using PRISMA or Cochrane methods), nor did we conduct any statistical summary methods such as meta-analysis. The included studies represent the consensus views and key evidence that define the contemporary role of NT-proBNP in clinical practice.

The age of precision medicine and evolving biomarkers for HF

**Figure 1:**
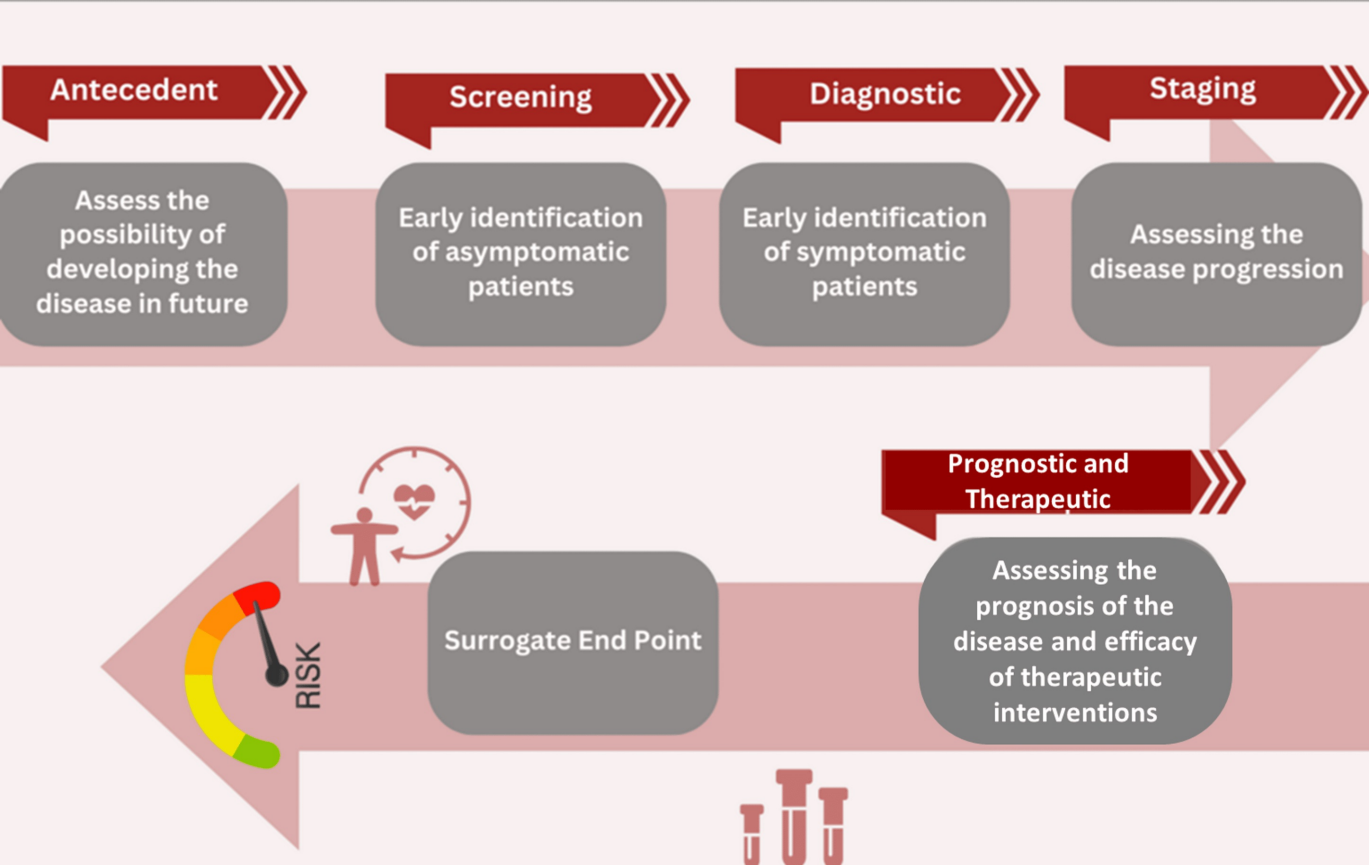
The newer biomarkers discovered over time for establishing the diagnosis and prognosis of heart failure This illustration categorizes different types of biomarkers - diagnostic, prognostic, predictive, pharmacodynamic, and monitoring - highlighting their distinct roles in disease detection, treatment selection, therapy response assessment, and patient management. It visually represents how biomarkers contribute to personalized medicine and improved clinical outcomes (Image created by the authors using Canva.com)

In the modern era of medicine, the ability to align disease mechanisms with individual genetic and environmental factors has paved the way for precision medicine. This approach integrates large-scale data on genetics, lifestyle, and clinical parameters to guide individualized therapy beyond traditional “signs and symptoms” [18]. Although biomarker use in precision medicine remains in its early stages, validation studies highlight their potential as real-time indicators of disease activity [19]. Figure 1 illustrates the key biomarker types and their clinical applications [20].

Biomarkers enhance the diagnosis and management of HF by reflecting distinct pathophysiological mechanisms in both HFrEF and HFpEF. NT-proBNP-guided therapy has been shown in randomized trials to reduce HF-related hospitalizations, while sST2 levels are increasingly used to tailor treatment intensity and monitor therapeutic response. In HFrEF, cardiac myocyte ischemia leads to fibrosis and remodeling, whereas HFpEF is characterized by comorbidity-driven inflammation and hypertrophic structural changes. The complex interplay of myocardial stretch, injury, inflammation, remodeling, fibrosis, and neurohormonal activation generates a spectrum of measurable biomarkers with diagnostic and prognostic significance [21]. The best-established include BNP, NT-proBNP, and high-sensitivity cardiac troponin (hs-cTn) [1]. Emerging markers-soluble suppression of tumorigenicity-2 (sST2), growth differentiation factor-15 (GDF-15), and galectin-3 (Gal-3)-are under active investigation for complementary prognostic value [22].

sST2 is released in response to myocardial stress and injury, providing independent prognostic information for mortality and hospitalization in both acute and chronic HF [23-25]. An FDA-cleared assay (Presage ST2) supports its use in risk stratification, though cost and assay accessibility currently limit widespread adoption. GDF-15 levels rise with cardiac inflammation and remodeling and correlate with adverse outcomes and worsening renal function in large observational studies [26-29]. However, assay variability and unclear tissue specificity have delayed FDA approval. Galectin-3, involved in macrophage activation and myocardial fibrosis, is FDA-approved as an adjunctive biomarker for chronic HF prognosis, though interpretation is confounded by elevations in renal disease, diabetes, and aging [30-32].

**Figure 2:**
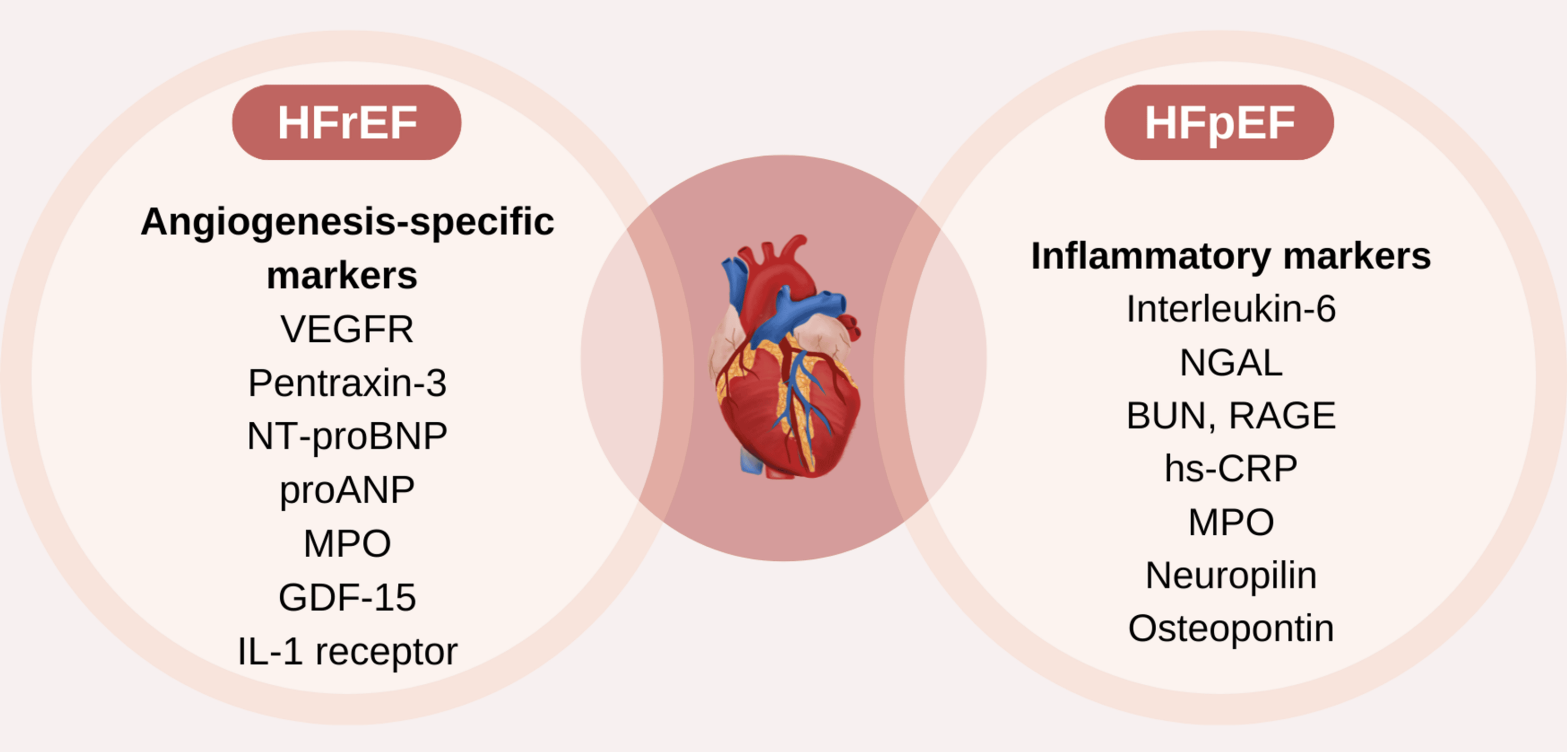
Biomarkers in HFrEF vs. HFpEF This illustration compares biomarkers associated with heart failure with reduced ejection fraction (HFrEF) and heart failure with preserved ejection fraction (HFpEF). HFrEF is linked to angiogenesis-specific markers such as VEGFR, NT-proBNP, and GDF-15, while HFpEF is associated with inflammatory markers like Interleukin-6, hs-CRP, and Osteopontin. The central heart image emphasizes the distinct yet interconnected pathophysiological mechanisms of both conditions. (Image created by the authors using Canva.com) VEGFR: vascular endothelial growth factor; NT-proBNP: N-terminal prohormone of brain natriuretic peptide; GDF-15: growth differentiation factor 15; proANP: pro atrial natriuretic peptide; MPO: myeloperoxidase; IL-1 receptor: interleukin-1 receptor; NGAL: renal marker neutrophil gelatinase-associated lipocalin; BUN: blood urea nitrogen; RAGE: inflammation marker receptor of advanced glycation end-products

Technical challenges-such as inter-assay variability, overlapping concentration ranges across HF subtypes, and comorbidity-related elevations-limit single-biomarker precision [33]. Consequently, multi-marker strategies combining NT-proBNP with markers of fibrosis or inflammation (e.g., sST2 or Gal-3) are emerging as a more accurate and personalized approach to HF phenotyping and risk prediction (Figure 2).

Association between EF category and CV versus non-CV events

The importance of EF status in HF treatment is widely acknowledged. A plethora of studies confirm the efficacy of drug and device interventions in reducing mortality and hospitalization rates among HFrEF patients. It is important to note that the success of these interventions is not mirrored in HFpEF treatment, as documented extensively [34-36].

Managing HFpEF can be a significant challenge as current treatment protocols are primarily tailored to HFrEF. This may be because HFpEF and HFmrEF are complex, systemic, and inflammatory diseases involving endothelial dysfunction and myocardial fibrosis, in contrast to the predominant contractile failure seen in HFrEF [37]. Therefore, the treatment of HFpEF requires a more comprehensive approach that addresses the underlying fibrotic and metabolic factors contributing to its development. Despite ongoing research efforts, there remains significant uncertainty surrounding the efficacy of various methods being investigated to reduce morbidity and mortality among patients with HFpEF. Although trials are currently underway, the results of these studies are still unknown and require further analysis and evaluation [34,35].

Patients afflicted with HFpEF and concurrent comorbidities such as obesity, hypertension, diabetes, and renal dysfunction face an elevated risk of hospitalization, particularly for non-HF-related causes, in contrast to their HFrEF counterparts [38]. Additionally, a decrease in LVEF is a strong predictor of adverse cardiovascular outcomes in patients with symptomatic heart failure, including all-cause mortality, cardiovascular mortality, sudden death, heart failure-related death, fatal or non-fatal myocardial infarction, and hospitalization related to heart failure [39]. Solomon et al.’s analysis of the CHARM program showed that with progressively declining EF, there is a significant, progressive increase in the risk of all-cause mortality, the most frequent causes being sudden cardiac death and HF-related death.

In contrast, it was found that the prognostic ability of ejection fraction is limited in patients with an LVEF above 45%, indicating that factors other than systolic function contribute to the risk of adverse outcomes in HFpEF [39]. Furthermore, other studies have demonstrated that comorbidities hold significant importance in these patients, with 30% of their mortality being non-cardiovascular in nature, despite suffering from symptomatic HF [38]. NT-proBNP retains prognostic utility across the EF spectrum, but its diagnostic specificity and interpretability are stronger in HFrEF. In HFpEF, confounding factors such as advanced age, obesity, and renal impairment can alter NT-proBNP levels, thereby limiting its diagnostic precision. 

NTproBNP and CV outcomes in HFpEF, HFmrEF, and HFrEF

**Figure 3:**
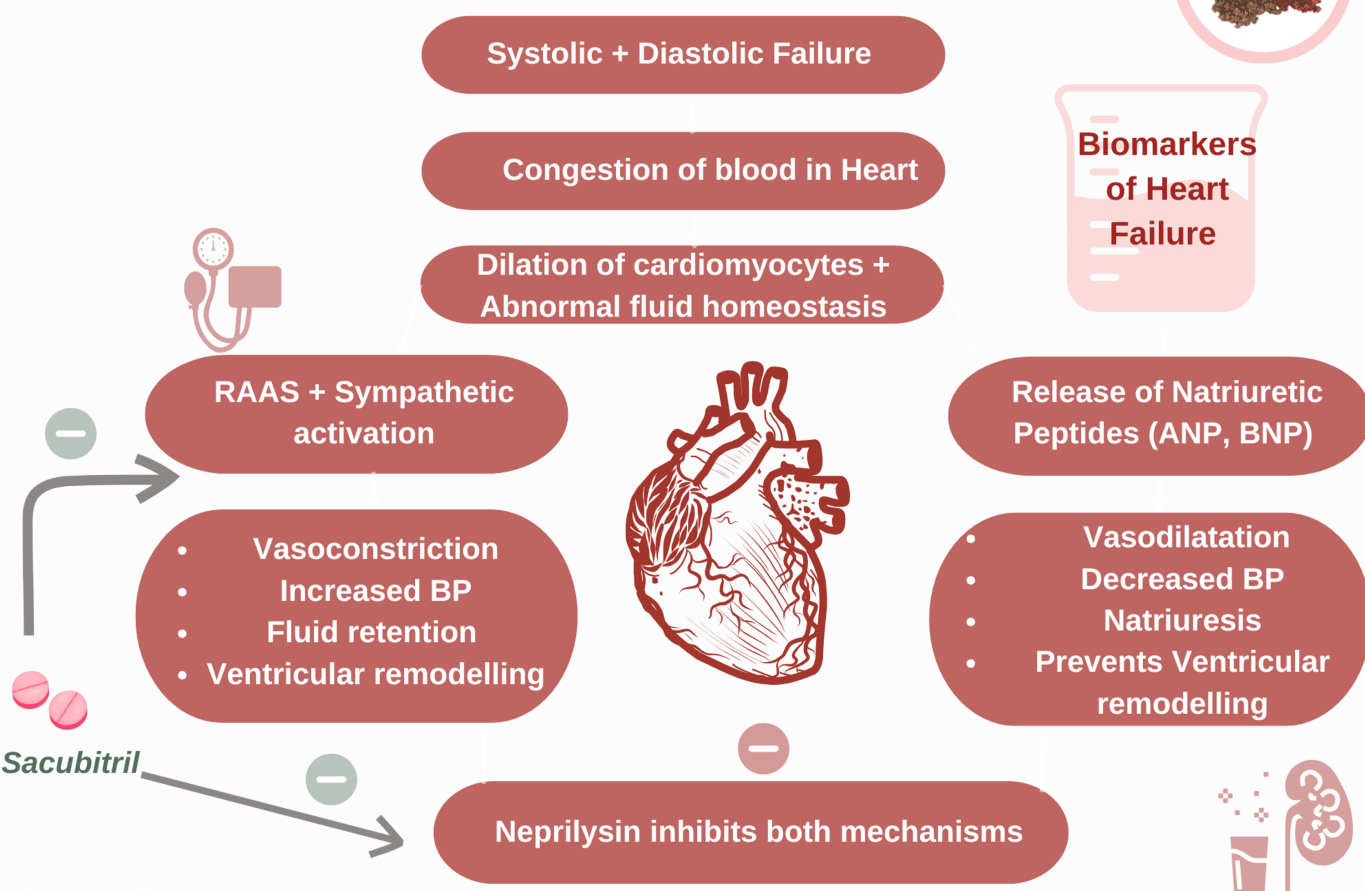
BNP and NTproBNP: role in the etiopathogenesis of heart failure This illustration depicts the dynamic interplay between natriuretic peptides and neurohormonal activation in heart failure. BNP and NT-proBNP are released in response to myocardial stretch and wall stress, promoting vasodilation, natriuresis, and inhibition of ventricular remodeling. In contrast, activation of the RAAS and sympathetic nervous system contributes to vasoconstriction, increased afterload, and fluid retention. Sacubitril, a neprilysin inhibitor, enhances BNP bioavailability by preventing its degradation, reflecting the therapeutic modulation of these pathways in HFrEF. The figure also highlights that the relative contribution of these mechanisms may differ across HF (Image created by the authors using Canva.com) RAAS: renin-angiotensin aldosterone system; BP: blood pressure; ANP: atrial natriuretic peptide; BNP: brain natriuretic peptide; NT-proBNP: N-terminal prohormone of brain natriuretic peptide

NTproBNP is a gold standard biomarker for the diagnosis and prognosis of heart failure. Figure 3 illustrates the pathophysiology associated with the rise in BNP and NTproBNP levels in patients with heart failure and the regulatory effect of neprilysin. A rise in its value increases the risk of onset or worsening of an already existing failure, irrespective of the ejection fraction [35]. Despite being the gold standard, it has certain drawbacks. The value of NTproBNP is lower and is influenced by various confounding factors in HFpEF and HFmrEF than in HFrEF. Therefore, it is more accurate when dealing with HFrEF [40]. As per the CHARM PROGRAM, left ventricular ejection fraction plays a key role in predicting the cardiovascular outcomes, fatal and nonfatal, including heart failure death, hospitalization, and fatal and nonfatal MI only in HFrEF, and therefore is a poor predictor in HFmrEF and HFpEF. Crude rates for CV events range from 20 to 160 per 100 patient-years in HFpEF; 20 to 130 in HFmrEF; and 20 to 110 in HFrEF, respectively [39].

Moreover, risk increased in all EF categories in patients with versus those without atrial fibrillation, ischemic heart disease, renal disease, and diabetes. Age ≥75 years versus <75 years and women versus men were associated with improved CV outcome in HFrEF but not in HFpEF and HFmrEF, even though no significant interaction between these subgroups and EF was reported. HF duration ≥6 months versus <6 months was associated with increased risk of CV events in HFmrEF and HFrEF, and of non-CV events only in HFrEF. Concluding, the risk of cardiovascular events is higher in patients with high NTproBNP levels, irrespective of ejection fractions, but the curve will be steeper in higher ejection fractions [35]. Finally, yet importantly, there are no disease-modifying therapies for HFpEF and HFmrEF like there are for HFrEF. Hence, the risk of cardiovascular events resulting in mortality is higher in the prior ejection fractions (Figure 3) [41].

NT-proBNP levels and prognosis of chronic HF: current guidelines

Elevated NT-proBNP levels reflect cardiac stress and correlate with HF severity [42,43], making them useful in assessing and managing cardiac health. Numerous studies have shown that NT-proBNP is a robust prognostic marker in chronic HF, predicting all-cause mortality and sudden death [44-47]. NT-proBNP has been reported as a better independent predictor of death than LVEF, RVEF, peak VO₂, and systolic blood pressure, and may serve as a marker for urgent cardiac transplantation in advanced HF [42]. Its prognostic value extends across HF phenotypes, including HFmrEF (EF 40-49%) and HFpEF (EF ≥50%) [45-49].

Short-term prognostic utility is evident before discharge in acutely decompensated HF, where elevated NT-proBNP predicts near-term mortality and readmission [50,51,52]. Long-term trends over 6-12 months correspond to both prognosis assessment and therapy titration, with reductions reflecting effective GDMT and improved outcomes [49,50,53-55]. For example, achieving NT-proBNP ≤1000 pg/mL within 90 days is associated with a 74% reduction in HF hospitalization or CV death and a 66% reduction in all-cause mortality [55].

Guideline-directed and observational studies indicate that a ≥30% rise in NT-proBNP is associated with worse outcomes, while a ≥30% reduction (or ≤45% in some studies) predicts improved prognosis [48,49]. These thresholds are derived from both guideline recommendations (e.g., AHA/ESC) and large observational cohorts, primarily in outpatient or chronic HF settings. Baseline NT-proBNP ranges vary across studies, but trends generally reflect relative changes from individualized baselines. In obese or overweight patients, absolute NT-proBNP values are lower, yet relative changes remain predictive, highlighting the need to interpret results alongside clinical context and co-morbidities such as renal impairment [54].

NT-proBNP retains predictive value regardless of AF status or HF phenotype [56], though interpretability is stronger in HFrEF. In HFpEF and HFmrEF, confounding factors such as age, obesity, renal dysfunction, and comorbidity burden may affect absolute levels, emphasizing the importance of serial measurements and trend evaluation rather than single thresholds [53]. Current practice guidelines recommend monitoring NT-proBNP at baseline, during therapy titration, and in select outpatient follow-ups to guide risk stratification and therapy adjustment, though frequent testing may be limited by cost and logistical considerations.

Association between NT-proBNP levels and prognosis (ESC guidelines)

NT-proBNP demonstrates independent prognostic power in patients with CHF of ischemic origin and in the development of heart failure following myocardial infarction [44]. NT-proBNP has been found to exhibit a superior ability to predict mortality independently when compared to traditional markers of heart failure, such as LVEF [57], peak oxygen consumption (VO_2_) [57], right ventricular ejection fraction (RVEF) [57], serum sodium levels [57], and systolic blood pressure [57]. NT-proBNP levels can be used to predict the risk of death or hospitalization in patients with heart failure, regardless of whether they have sinus rhythm or atrial fibrillation [58]. NT-proBNP was clearly able to assign a poor survival with far greater power [58].

NT-proBNP holds significant potential in determining which advanced heart failure patients may benefit from cardiac transplantation, surpassing the reliability of single severity parameters like the Heart Failure Survival Score (HFSS) [44]. With 15-20% of patients experiencing mortality within one year post-transplantation, higher NT-proBNP levels serve as a valuable predictor of death risk [44]. Patients with elevated NT-proBNP levels were four times more likely to die within the first year after transplantation, and the majority of deaths and adverse events occurred in this group. Hence, NT-proBNP levels can aid in identifying patients at a higher risk of mortality, guiding careful consideration for heart transplantation. However, further research is needed to evaluate its clinical application in larger patient populations referred for transplantation [44].

NT-proBNP may be a more useful marker of prognosis than BNP, especially if future treatment of congestive heart failure (CHF) is routinely used in clinical practice [44]. NT-proBNP serves as a valuable prognostic tool not only for patients with CHF, but also for individuals with other cardiac disorders such as myocardial infarction, valvular heart disease, atrial fibrillation, or pulmonary embolism [44]. NT-proBNP concentrations have high prognostic accuracy for death [58] and HF hospitalization [44].

The clinical efficacy of NT-proBNP is greatly influenced by confounding factors. Age nearly doubles NT-proBNP values with each decade, irrespective of sex and presence of heart disease [59]. In patients above 70 years with poor renal function (eGFR <45 mL/min/1.73m), the diagnostic ability of NT-proBNP decreases [60,61]. However, prognostic accuracy remains similar irrespective of age, suggesting higher cutoffs may be appropriate in older patients [62,63]. Females generally have higher NT-proBNP levels than males, and healthy women on hormone replacement therapy show elevated levels, suggesting a potential role of estrogen [64,65]. The prognostic power of NT-proBNP for long-term mortality and hospital readmissions has been reported to be higher in men than women [66].

Obesity also affects NT-proBNP. In a study of 39,937 individuals, obesity was associated with lower NT-proBNP levels in women compared to men [67]. Several studies report an inverse relationship between BMI and NT-proBNP in healthy subjects and HF patients [68-70]. Frankenstein et al. found a 4% drop in NT-proBNP per unit increase in BMI after matching for age, sex, and renal function [69]. Lower thresholds for HF diagnosis in obese populations have been suggested [71], though recent work indicates prognostic power remains intact up to BMI 40 kg/m² [72]. Renal impairment and anemia also elevate NT-proBNP independent of HF severity [73]. High plasma creatinine is independently associated with increased NT-proBNP, likely due to reduced clearance and increased LV wall stress [59]. The PRIDE study reported decreased sensitivity and specificity of NT-proBNP in HF diagnosis among patients with renal failure [51].

Certain HF medications, such as neprilysin inhibitors, can confound BNP measurements since natriuretic peptides are neprilysin substrates. NT-proBNP is preferred in these situations because it is not affected by neprilysin inhibition [74]. Clinical trials like EMPEROR and CANDLE showed that empagliflozin modestly reduced NT-proBNP in HFpEF patients [75-76], while EMPEROR-Reduced demonstrated a 5-13% reduction in HFrEF, which had greater prognostic significance than baseline values [77].

Overall, NT-proBNP provides powerful prognostic information but should be interpreted alongside clinical scores, imaging, and functional assessments, particularly in transplant decision-making. Patient factors, comorbidities, and therapies collectively require individualized NT-proBNP thresholds to guide risk stratification and management [44,51,57-77].

Regional variations and the role of genomics

NT-proBNP levels exhibit significant variability across different populations and regions, influenced by a complex interplay of factors including age, gender, co-morbidities, and genetic predisposition. Studies conducted in Europe, North America, and Asia provide valuable insights, although these findings may not be generalizable across all ethnicities or healthcare settings due to environmental, dietary, or systemic differences.

From a European perspective, a comprehensive study of the Scottish population by Welsh et al. revealed significant age- and gender-related differences in NT-proBNP levels. Participants were divided into three age groups (<30, 50-59, and ≥80 years), and a dramatic increase in NT-proBNP levels with age was observed in both genders. In males, median levels rose from 21 ng/L in the youngest group to 281 ng/L in the oldest, while in females, the increase was from 51 ng/L to 240 ng/L. The prevalence of elevated NT-proBNP (≥125 pg/mL) increased substantially with age, and after adjusting for risk factors, elevated NT-proBNP was more common in females. Older age and smoking were more strongly associated with elevated NT-proBNP in males, whereas diabetes showed an inverse association in females [78].

In North America, a U.S. cohort study found that higher NT-proBNP levels were associated with older age, female sex, higher blood pressure, lower eGFR, and higher HbA1c. Comorbidities such as hypertension, diabetes, and albuminuria were more prevalent in participants with higher NT-proBNP levels, while smoking was less common, and no significant association with BMI was observed [79]. From an Asian perspective, a Turkish study categorized participants by NT-proBNP quartiles, showing higher quartiles associated with female sex, older age, and increased prevalence of diabetes, hypertension, and coronary artery disease. Creatinine clearance, total cholesterol, and triglycerides decreased progressively with increasing NT-proBNP quartiles [80].

Genetic studies further illuminate the hereditary contributions to NT-proBNP variability. Xhaard et al. (2022) in the STANISLAS cohort estimated that ~15% of plasma NT-proBNP variation is attributable to genetic factors, identifying SNPs in NPPA/NPPB, CLCN6, and MTHFR. Certain NPPB-NPPA SNPs correlated with diastolic and metabolic functions, suggesting potential applications in personalized NT-proBNP thresholds or predictive algorithms [81]. Similarly, Johansson et al. (2016) identified three SNP alleles associated with elevated NT-proBNP, highlighting modest gene-by-environment interactions [82]. These findings reinforce that while NT-proBNP remains a valuable biomarker, genetic and population-specific factors may necessitate individualized interpretation.

Comparative NT-proBNP cutoff values for diagnosis or prognosis differ regionally, and current universal cutoffs (e.g., ≥125 pg/mL for chronic HF, >300 pg/mL for acute HF) [82] may require adjustment in the context of atrial fibrillation, obesity, or renal impairment. For example, thresholds in AF patients may need to be approximately threefold higher than in sinus rhythm, whereas obesity may result in lower circulating levels, increasing the risk of false negatives. Combined biomarker strategies incorporating NT-proBNP with IGFBP-7, troponins, or sST2 may enhance diagnostic accuracy, though cost, assay availability, and clinical feasibility remain limiting factors.

Finally, the reported NT-proBNP rise after 48 weeks of sacubitril/valsartan therapy (e.g., PROVE-HF and PARAGON-HF) was generally modest, clinically non-significant, and potentially related to assay variability or physiological adaptation, rather than indicative of worsening HF. These observations emphasize that NT-proBNP should not be used in isolation, particularly in HFpEF, and underscore the need for a multimodal, individualized diagnostic approach.

Challenges in employing NT-proBNP for HFpEF diagnosis

Diagnosing HFpEF is a huge predicament for the clinician. Unlike HFrEF, it does not have definite echocardiographic parameters. Emergence of circulating biomarkers like BNP and NTproBNP has proven to be a boon. However, its use in HFpEF is flawed as the range of NTproBNP can sometimes extend down to a normal value in some patients, therefore, hampering a clear idea of the risk. Moreover, it has been seen in one of the current studies that the initial reduction in NTproBNP levels after using sacubitril/ valsartan was followed by a rise in its values after 48 weeks of follow-up. A similar trend was observed in the PROVE-HF study. One of the assumptions for this disparity is assumed to be the various confounding factors, like atrial fibrillation, renal impairment, and obesity [40].

In addition to this, the value of NTproBNP is lower in HFpEF as compared to HFrEF. A rising trend is observed in patients with atrial fibrillation, supported by the PARAGON-HF trial, which considered a three-fold higher NTproBNP threshold in cases of atrial fibrillation to achieve a comparable risk assessment to that of patients in sinus rhythm [83]. Furthermore, a similar increase in threshold was considered in patients with impaired renal function. As opposed to the above factors, a downward spiral is seen in obese patients. In addition to the already low values in HFpEF as compared to HFrEF, a further fall in obese individuals can lead to underdiagnosis [84]. The above disparities have made this simple biomarker strategy inadequate. Hence, a combined biomarker strategy is now being considered employing biomarkers like cardiac troponins and insulin-like growth factor binding protein-7 [40].

Natriuretic peptide cut-off points

BNP and NT-proBNP are well-established diagnostic and screening markers for HF. Gaggin et al. (2015) provide comprehensive cut-off points that aid clinical assessment [85]. For acute dyspnea, BNP levels below 30-50 pg/mL and NT-proBNP below 300 pg/mL effectively exclude acute HF, while single cut-offs of BNP ≥100 pg/mL or NT-proBNP ≥900 pg/mL indicate likely acute HF. A “grey zone” of BNP 100-400 pg/mL requires further evaluation. For outpatient screening, thresholds are age- and symptom-dependent: asymptomatic individuals may use BNP <20 pg/mL and symptomatic individuals <40 pg/mL; NT-proBNP cut-offs are <125 pg/mL for patients under 75 years and <450 pg/mL for those ≥75 years. Januzzi et al. proposed age-specific NT-proBNP thresholds (≥450 pg/mL for <50 years, ≥900 pg/mL for 50-75 years, and ≥1800 pg/mL for >75 years) with optimal diagnostic performance [86]. Pan et al. suggested a cut-off of 257.4 ng/L for chronic HF, showing improved predictive value [87-91]. These cut-offs balance sensitivity and specificity, emphasizing that natriuretic peptides support but do not replace clinical judgment.

NT-proBNP-directed HF therapy and cardiovascular vs. non-cardiovascular outcomes

The dynamic nature of NT-proBNP allows it to guide goal-directed therapy, including up-titration of guideline-directed medical therapy (GDMT) and earlier interventions [92]. In PIONEER-HF, NT-proBNP-guided therapy in hospitalized, stabilized patients improved outcomes; however, long-term outcome comparisons between in-hospital versus post-discharge initiation remain limited, particularly regarding readmission risk and therapy responsiveness [93].

Elevated NT-proBNP is associated with both cardiovascular (CV) and non-CV events. For instance, patients with HFrEF have a higher ratio of CV to non-CV events (~1.5), reflecting pump failure, while HFpEF patients have lower CV/non-CV ratios (~2/3), reflecting a greater comorbidity burden (e.g., liver disease, COPD, anemia, diabetes, CKD) [1,35,38]. Atrial fibrillation acutely raises NT-proBNP, peaking within 24-36 hours, after which levels decline even if AF persists [94]. These NT-proBNP trends can guide therapy titration, such as GDMT up-titration or discharge planning.

Evidence for NT-proBNP influencing non-CV outcomes is limited; mechanistically, reductions in NT-proBNP via effective therapy may indirectly improve organ perfusion, renal function, or hemodynamic stress, though robust outcome data are lacking [95]. Therefore, NT-proBNP primarily serves as a prognostic biomarker, while its effect on non-CV outcomes should be interpreted cautiously.

Limitations

We have made diligent efforts to incorporate the most recent and updated data and studies available in our review. However, it is important to acknowledge that, due to the nature of a narrative literature review and the absence of a predefined selection strategy - including specific search methodology and formal inclusion/exclusion criteria - the possibility of selection bias cannot be ruled out. Inherent bias in narrative reviews may influence which studies are included, independent of individual author opinions. We also acknowledge that we have included only published studies, which could potentially lead to publication bias and omission of statistically significant data or other advancements reported in unpublished studies. This may limit the comprehensiveness of our review. We have attempted to include studies with participants from diverse regions, races, ethnic backgrounds, and other relevant factors to enhance applicability to a broader population. Nevertheless, certain groups may remain underrepresented-for example, individuals from low-income regions, ethnic minorities, and pediatric patients with heart failure-potentially limiting generalizability. Therefore, these limitations should be considered when interpreting our findings, and further research, including systematic reviews and meta-analyses, is warranted. 

Future directions

NT-proBNP levels have been extensively employed as an inclusion standard in clinical trials and as substitute measures within research to assess how treatment affects this biomarker and its correlation with overall results (Table 1). Given that patient characteristics, trial design, treatment mechanisms, or other variables could influence the connection between natriuretic peptides and outcomes, gaining deeper insights into these associations is essential for accurately interpreting interventions and their effects on outcomes [96].

**Table 1:** Overview of existing clinical evidence for NT-pro-BNP* ^*^[97-100] NT-pro-BNP: N-terminal proBNP; HF: heart failure; LVEF: left ventricular ejection fraction; MI: myocardial infarction; DM: diabetes mellitus; HTN: hypertension; CI confidence interval

Clinical trial number	Trial title	Type of study	Stage of trial	Sample size/type	Disease studied	Results	
NCT0341221	STRONG-HF (Safety, Tolerability, and Efficacy of Up-titration of Guideline-Directed Medical Therapies for Acute HF)	Multicenter, randomized, parallel group study with no masking (open-label)	Phase 3	1077 - humans	Acute HF and a >10% NT-proBNP decrease from screening (i.e., admission) to randomization (i.e., pre-discharge) were included	Among patients with acute HF enrolled in STRONG-HF, high-intensity care (HIC) reduced the risk of 180-day HF readmission or death, regardless of baseline NT-proBNP. GDMT (guideline-directed medical therapy) up-titration early post-discharge, utilizing increased NT-proBNP as guidance to increase diuretic therapy and reduce the GDMT up-titration rate, resulted in the same 180-day outcomes regardless of early post-discharge NT-proBNP change	Adamo et al. (2023) [97]
NCT02924727	PARADISE MI (Prospective ARNI vs. ACE Inhibitors Trial to Determine Superiority in Reducing Heart Failure Events After MI)	Multi-centre, active-controlled, randomized parallel assignment with quadruple masking (participant, care provider, investigator, and outcomes assessor)	Phase 3	1129 (substudy of original sample size of 5661) - humans	Spontaneous acute myocardial infarction along with LVEF ≤40% after index MI presentation and before randomization and/or pulmonary congestion requiring intravenous treatment with diuretics, vasodilators, vasopressors, and/or inotropes, during the index hospitalization	Patients in the highest quartile of NT-proBNP were older, more commonly women, and had more hypertension, atrial fibrillation, renal dysfunction, and pulmonary congestion on presentation. (all p<0.001). NT-proBNP was strongly associated with the primary end point (adjusted hazard ratio, 1.45 per doubling of NT-proBNP; [95% CI, 1.23-1.70]), adjusted for clinical variables and baseline hs-cTnT. NT-proBNP was also independently associated with all-cause death (adjusted hazard ratio, 1.74 [95% CI, 1.38-2.21]) and fatal or nonfatal MI or stroke (adjusted hazard ratio, 1.24 [95% CI, 1.05-1.45])	Jering et al. (2023) [98]
NCT02036450	LOOP Study (Atrial Fibrillation Detected by Continuous ECG Monitoring Using Implantable Loop Recorder (ILR) to Prevent Stroke in High-Risk Individuals)	Interventional randomized parallel assignment with no masking (open-label)	Not applicable	5819 (96.9% of the trial population of 6004) - humans	Previous H/O of stroke/HF/DM/HTN	In an older population with additional stroke risk factors, ILR screening for AF was associated with a significant reduction in stroke risk among individuals with higher NT-proBNP levels but not among those with lower levels	Xing et al. (2023) [99]
NCT01106014	GRIPHON trial (Prostacyclin [PGI_2_] Receptor Agonist In Pulmonary Arterial Hypertension)	Multicentre, placebo-controlled, randomized parallel assignment with quadruple masking. (participant, care provider, investigator, and outcomes assessor)	Phase 3	1156 - humans	Pulmonary artery hypertension	Baseline and follow-up NT-proBNP categories were highly prognostic for future morbidity/mortality events during the study. Selexipag reduced the risk of morbidity/mortality events across all 3 NT-proBNP categories in both the baseline and time-dependent analyses, with a more pronounced treatment benefit of selexipag seen in the medium and low NT-proBNP subgroups	Chin et al. (2019) [100]

## Conclusions

HF manifests through a spectrum of symptoms and clinical signs and remains a major global health crisis, primarily attributed to coronary artery disease. Affecting over 37 million people worldwide, its prevalence is particularly high among the elderly. HF is classified into HFrEF, HFpEF, and HFmrEF. The pathophysiology of these subtypes involves varying mechanisms of myocardial damage and dysfunction, contributing to elevated mortality and significant healthcare burdens. This review highlights the diagnostic and prognostic value of BNP and NT-proBNP in managing HF, focusing on their roles across ejection fraction categories, treatment implications, and patient outcomes. NT-proBNP levels are strongly associated with cardiovascular outcomes and mortality risk, though interpretation remains constrained in subgroups such as HFpEF, obese, or elderly patients, and those with renal dysfunction. Clinicians should integrate NT-proBNP with clinical assessment, imaging, and comorbidity profiles for optimal use in current practice. Novel biomarkers such as soluble ST2 and GDF-15 offer additional insights to refine HF management. While NT-proBNP remains the gold standard, understanding its limitations is essential. Future directions include the development of ejection fraction-specific biomarker algorithms and AI-assisted NT-proBNP interpretation models to personalize HF management and improve outcomes in diverse patient populations.
